# NHC-catalysed highly selective aerobic oxidation of nonactivated aldehydes

**DOI:** 10.3762/bjoc.9.65

**Published:** 2013-03-22

**Authors:** Lennart Möhlmann, Stefan Ludwig, Siegfried Blechert

**Affiliations:** 1 TU-Berlin – Berlin Institute of Technology, Institute of Chemistry, Straße des 17. Juni 115, 10623 Berlin, Germany. Fax: (+49)-30-314-29745; Tel:(+49)-30-314-22255

**Keywords:** aerobic oxidation, chemoselective oxidation, metal-free oxidation, *N*-heterocyclic carbene, organocatalysis

## Abstract

This publication describes a highly selective oxidation of aldehydes to the corresponding acids or esters. The reaction proceeds under metal-free conditions by using *N*-heterocyclic carbenes as organocatalysts in combination with environmentally friendly oxygen as the terminal oxidation agent.

## Introduction

The development of efficient and selective aerobic oxidation methods for organic substrates is of increasing interest and an attractive goal in green chemistry [1–3]. In a recent communication we reported on selective oxidation of carbinols to aldehydes or ketones using oxygen, visible light and mesoporous graphitic carbon nitride (mpg-C_3_N_4_) polymer as a metal-free photocatalyst [4]. As an extension of this method we were interested in a consecutive organocatalytic process using an *N*-heterocyclic carbene (NHC) together with mpg-C_3_N_4_ and oxygen.

**Scheme 1:**
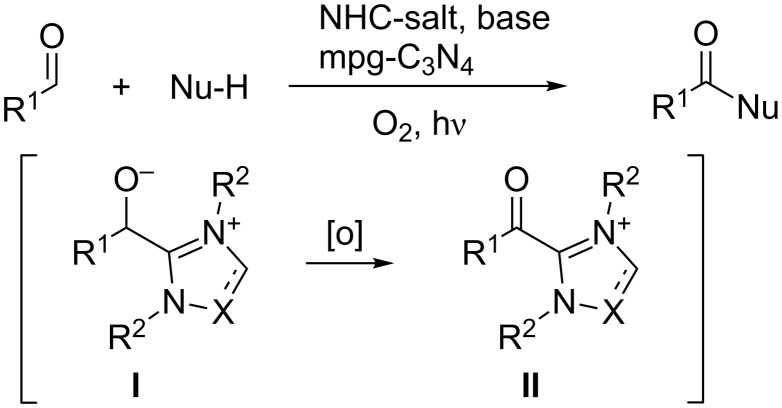
NHC/mpg-C_3_N_4_-catalysed aerobic oxidation of aldehydes.

NHC-catalysts derived from the corresponding salts are known to react with aldehydes to provide **I** and lead after oxidation to acyl cation intermediate **II**. Subsequent reactions with nucleophiles give esters, amides or acids. Reagents such as MnO_2_ [5–7], DDQ, TEMPO and azobenzene [8–18] were described. The use of mpg-C_3_N_4_ should lead to a new process containing two subsequent organocatalytic reactions (Scheme 1).

## Results and Discussion

**Scheme 2:**
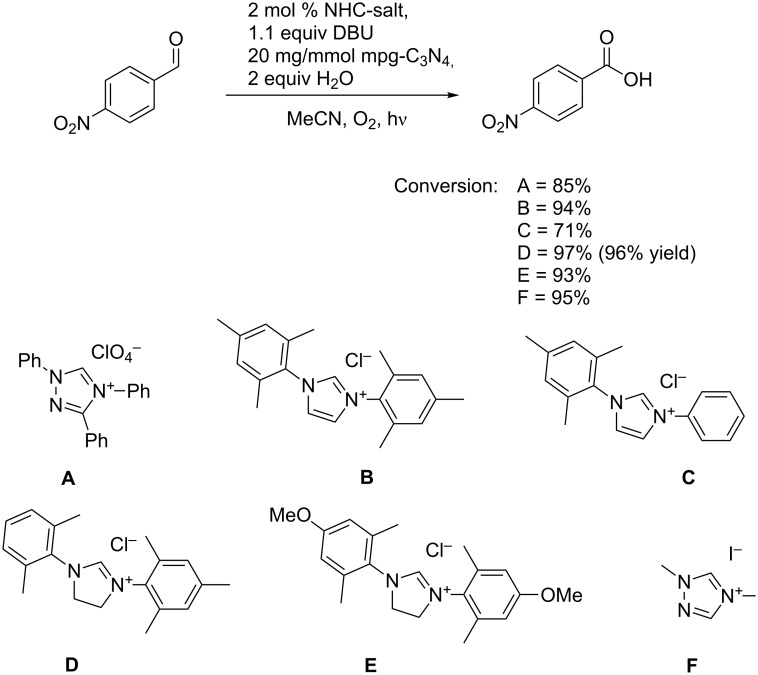
Oxidation of 4-nitrobenzaldehyde with different NHC-salts as precatalysts.

In initial experiments we used 4-nitrobenzaldehyde as a model substrate. As NHC-salt we selected **A**, which is known to yield highly stable carbenes [19]. Using acetonitrile as solvent, 2 equivalents of water as a nucleophile, 2 mol % **A**, 1.1 equivalents of DBU and 20 mg/mmol mpg-C_3_N_4_, we obtained after 1 h under an O_2_ atmosphere, 85% conversion to the wanted acid. Next we tested different NHC-salts **B**–**E** as precatalysts, varying the sterics and electron densities (Scheme 2) [19–22]. All carbenes were reactive; however, the salt **D** gave the best results. With 2 mol % **D** we obtained 97% conversion (96% isolated product). With 0.5 mol % the reaction needed 2 h for the same yield. With the best NHC catalyst in hand, we tried oxidation reactions of cyclohexanecarbaldehyde as a nonactivated substrate.

During these studies we observed a catalytic oxidation with oxygen using 20 mg mpg-C_3_N_4_ per millimol of aldehyde, even without light. Following the reaction by GC-analysis showed an exponential decrease of the reaction rate.

**Figure 1:**
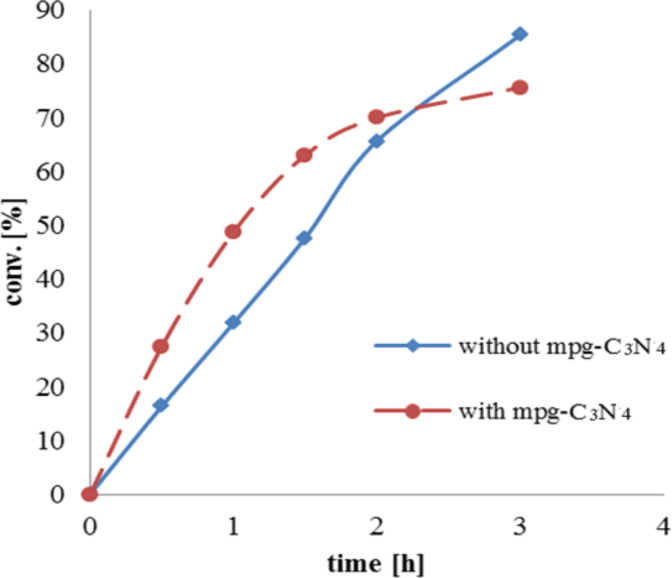
Oxidation of cyclohexanecarbaldehyde to cyclohexanecarboxylic acid under aerobic conditions with and without mpg-C_3_N_4_.

Testing the dependence of the reaction on the amount of mpg-C_3_N_4_ catalyst, we also observed the formation of acid even in the absence of this catalyst but with significantly slower conversion during the first two hours, i.e., after 0.5 h, 16% conversion and after 1 h, 32%, whereas in the presence of mpg-C_3_N_4_ (20 mg/mmol) the respective conversions were enhanced by a factor of 1.7 to 27% and by a factor of 1.5 to 49%, (see Figure 1).

As shown, the accelerating effect decreases over time and the reaction is finished after 3 h. Finally, the reaction without the second catalyst led to a higher total conversion. We suppose that this is caused by the oxidation of the NHC, i.e., the consumption of the umpolung catalyst [23].

**Figure 2:**
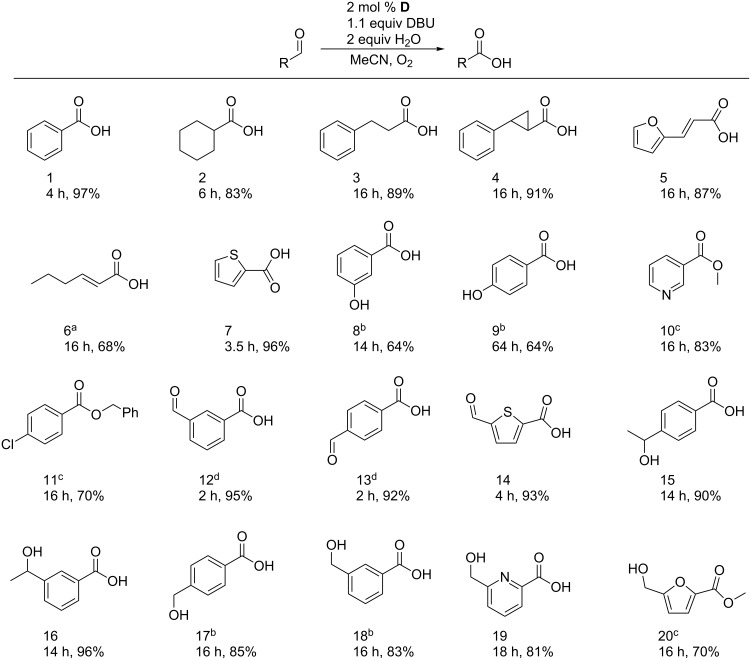
Oxidation of various aldehydes under the optimized conditions: ^a^2 equiv K_2_CO_3_ was used instead of DBU; ^b^10 mol % of **D** was used; ^c^5 equiv of the corresponding alcohol were used instead of H_2_O; ^d^0.5 mol % of **D** was used. The data below the products refer to the reaction times and the yields of the corresponding transformations.

With this result in hand we focused on the aerobic autoxidation using only **D** as a precatalyst. An interesting conversion of aromatic aldehydes to acids with 15 mol % of an NHC-salt, DBU and CO_2_ has been reported by Nair et al. [24]. However Bode et al. recently concluded that oxygen is the oxidant in these reactions [25]. An oxidative carboxylation of activated arylaldehydes with 10% water in DMF by 5 mol % of a sulfoxylalkyl-substituted NHC has been described by Yoshida et al. [26]. With electron-deficient arylaldehydes the yields are good to moderate. However, benzaldehyde itself gave less than 10% product. Under our conditions using 2 mol % **D** and 2 equiv H_2_O we obtained benzoic acid with a yield of 97%. Encouraged by this result, we carried out further systematic studies that have not previously been done, to gain more information about the substrate scope and especially the selectivity of this NHC-catalysed aerobic oxidation. The results are summarized in Figure 2.

To our delight, various aldehydes, including aliphatic (Figure 2, entries 2–4), α,β-unsaturated (Figure 2, entries 5 and 6) and electron-rich (Figure 2, entries 7–9) substrates, could be converted to the corresponding acids in good to excellent yields. It is worth mentioning that the use of air instead of oxygen increases the reaction times slightly, but does not decrease the yields. We assume that the oxygen concentration in the reaction solution is relatively similar due to the rather low solubility of O_2_ in organic solvents. However, vigorously shaking or stirring is essential. For hydroxy-substituted benzaldehydes only moderate yields (64%) could be achieved, even with enhanced catalyst loading; however, aerobic oxidations of these highly electron-rich compounds are barely described in the literature, and to the best of our knowledge this is the first metal-free example that even proceeds at room temperature. (Examples for metal-catalyzed aerobic oxidations can be found in [27–28].) The methodology could be extended to oxidative esterification by using alcohols as nucleophiles instead of water. However, this transformation is highly sensitive to moisture, which leads to the formation of acid as a side product. Even under dry conditions, with freshly distilled solvents, and by adding molecular sieves to the reaction solution we were not able to inhibit the acid formation totally, and thus, the yields are slightly reduced (Figure 2, entries 10, 11 and 20).

After demonstrating the versatility of this oxidation we further investigated the application of this very mild method for more interesting chemoselective transformations. Therefore, several substrates were oxidised under our conditions bearing either a second alcohol or aldehyde functionality. Desymmetrization of dialdehydes by selective oxidation is an important challenge, since symmetric dialdehydes are often easy to synthesize and/or are commercially available, and are thereby popular building blocks [29]. Pleasingly, our protocol provided excellent yields in short reaction times for all tested substrates (Figure 2, entries 12–14).

The selective oxidation of aldehydes in the presence of activated alcohol functions is a demanding task, since concurrent oxidation of the alcohol can occur easily [30–31]. The mild conditions of this NHC-catalysed aerobic oxidation, however, allow the specific transformation of the aldehyde moiety (Figure 2, entries 15–20) in good to excellent yields to the desired products. For entry 17 and 18 the oxidation occurred at a slower rate, and it was necessary to increase the catalyst loading to get a sufficient conversion.

These kinds of transformations are of great importance not only for academic purposes but also for industrial applications. HMF (Figure 2, entry 20) for example can nowadays be gained directly from the conversion of biomass and thereby became an attractive synthesis unit in modern "green" chemistry [32]. Hence, further cheap and environmentally friendly selective transformations are of great interest. However, only a few methods have been described for the selective oxidation of HMF, and all rely on the use of expensive noble metals at high temperatures, which makes an industrial application unbecoming [31,33–35]. Pleasingly, following our protocol we were able to oxidise this compound selectively to the methyl ester in good yield (70% after purification).

**Scheme 3:**
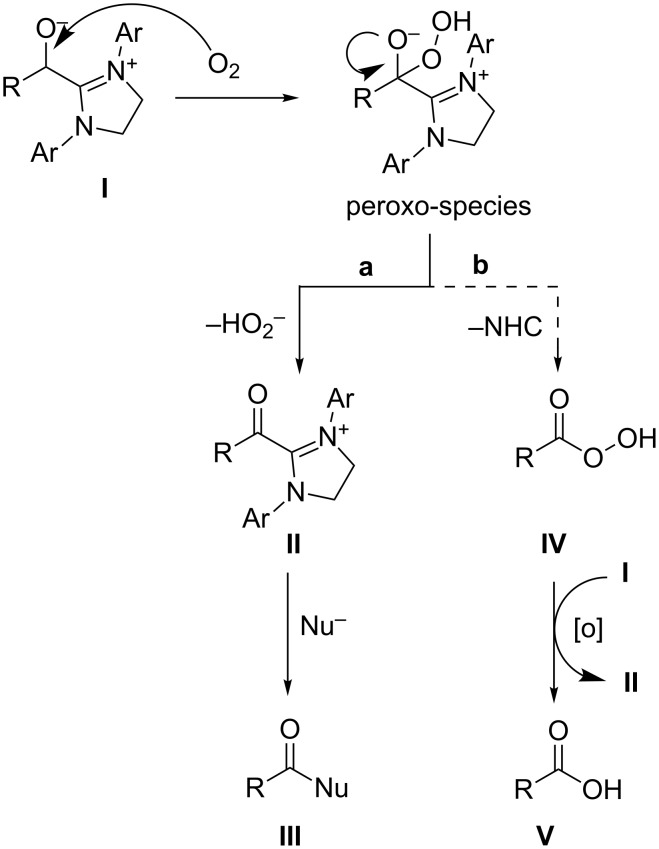
Proposed mechanism for the aerobic oxidation.

Our proposed mechanism for the aerobic oxidation is presented in Scheme 3. The highly activated intermediate **I**, which is formed by the addition of the NHC to the aldehyde, reacts with dioxygen to form the peroxo-species [36–37].

This unstable compound can now decompose into two different products. Pathway **a** would be the generation of the active ester **II** by abstraction of HO_2_^−^. In the presence of a nucleophile, **II** will then form the final product (ester or acid) under regeneration of the NHC. A second possible pathway **b** is the formation of the peroxy acid **IV** by abstraction of the NHC. **IV** is a strong oxidation agent itself and thereby able to oxidise a second molecule of **I** under formation of acid **V** and active ester **II**. Again **II** will then react with a corresponding nucleophile to furnish the product **III**. An additional reaction, which cannot be excluded, is that **IV** reacts directly with the aldehyde to form two molecules of **V**.

Based on our results we believe that pathway **a** is favoured for this reaction since **b** could only deliver a maximum of 50% of the oxidative esterification product in the presence of an alcohol as a nucleophile. In our experiments, however, yields up to 83% of the desired ester were achieved. Following path **a**, a hydroperoxyl anion is formed as a side product. In our experiments we could not detect this anion. We therefore assume that it acts as an additional oxidation agent under formation of water.

To fortify this prediction, a test experiment with H_2_O_2_ as the terminal oxidation agent instead of O_2_ under nitrogen atmosphere was carried out for the NHC-catalysed oxidation of 4-nitrobenzaldehyde to 4-nitrobenzoic acid. It revealed that the peroxide was capable of effecting the oxidation as well providing comparable conversion.

## Conclusion

In summary we have developed an NHC-catalysed aerobic oxidation protocol, which is widely deployable to afford good to excellent yields. The reaction occurs under metal-free conditions at room temperature with low catalyst loadings and reasonable reaction times, demonstrating the desirability of this oxidation both from the economic as well as from the environmental point of view. Remarkably, highly selective transformations could be achieved as well as the direct esterification of aldehydes making this method a powerful tool in organic synthesis.

## Experimental Section

### General procedure for the aerobic oxidation

In a 10 mL Schlenk tube 0.5 ml MeCN, aldehyde (0.5 mmol), 1.1 equiv DBU, 2 mol % NHC and 2–5 equiv nucleophile (water or alcohol) were subsequently added. An O_2_ balloon was installed at the tube to provide an oxygen atmosphere. The solution was then shaken at room temperature until all starting material had been consumed. The reaction was quenched and worked up either by the addition of 2 M HCl solution (2 mL) followed by acidic and basic extraction or by removal of the solvent in vacuo followed by column chromatography on SiO_2_. Commercially available aldehydes were used without further purification. Nonavailable substrates were prepared as described in the literature. All products are literature-known and/or commercially available. The NMR data of all isolated products match those reported.
